# Author Correction: Engineering self-deliverable ribonucleoproteins for genome editing in the brain

**DOI:** 10.1038/s41467-024-48087-6

**Published:** 2024-05-01

**Authors:** Kai Chen, Elizabeth C. Stahl, Min Hyung Kang, Bryant Xu, Ryan Allen, Marena Trinidad, Jennifer A. Doudna

**Affiliations:** 1 Department of Molecular and Cell Biology, University of California, Berkeley, Berkeley, CA USA; 2 Innovative Genomics Institute, University of California, Berkeley, CA USA; 3 California Institute for Quantitative Biosciences, University of California, Berkeley, Berkeley, CA USA; 4 Howard Hughes Medical Institute, University of California, Berkeley, Berkeley, CA USA; 5 Gladstone Institutes, San Francisco, CA USA; 6 Gladstone-UCSF Institute of Genomic Immunology, San Francisco, CA USA; 7 Molecular Biophysics and Integrated Bioimaging Division, Lawrence Berkeley National Laboratory, Berkeley, CA USA; 8 Department of Chemistry, University of California, Berkeley, Berkeley, CA USA

**Keywords:** Biotechnology, Drug delivery, CRISPR-Cas9 genome editing, Genetic engineering, Peptides

Correction to: *Nature Communications* 10.1038/s41467-024-45998-2, published online 26 February 2024

In the Acknowledgements section of this article, the grant number relating to National Institutes of Health funding to J.A.D. was incorrectly given as RM1HG009490 and should have been U19NS132303. The grant number 2334028 relating to the National Science Foundation funding to J.A.D. was omitted. Funding from Hampton University Summer Undergraduate Research Program, Mr. Li Ka Shing, Emerson Collective and the Innovative Genomics Institute (IGI) were omitted. The original article has been corrected.

