# Supplementary material for: Engineering self-deliverable ribonucleoproteins for genome editing in the brain
Source: Nat Commun. 2024 Feb 26;15:1727. doi: 10.1038/s41467-024-45998-2 (PMC10897210; doi:10.1038/s41467-024-45998-2)
Supplement: Supplementary file 1 — Supplementary Information [file 41467_2024_45998_MOESM1_ESM.pdf]

Supplementary Materials

for

Engineering self-deliverable ribonucleoproteins for genome editing in the brain

Kai Chen[1,2†], Elizabeth C. Stahl[1-3†], Min Hyung Kang[1,2,4], Bryant Xu[1,2], Ryan Allen[1,2], Marena Trinidad[1,2,4], Jennifer A. Doudna[1-8*]

[1]Department of Molecular and Cell Biology, University of California, Berkeley; Berkeley, CA, USA.

[2]Innovative Genomics Institute; University of California, Berkeley, CA, USA.

[3]California Institute for Quantitative Biosciences, University of California, Berkeley; Berkeley, CA, USA.

[4]Howard Hughes Medical Institute, University of California, Berkeley; Berkeley CA, USA.

[5]Gladstone Institutes; San Francisco, CA, USA.

[6]Gladstone-UCSF Institute of Genomic Immunology; San Francisco, CA, USA.

[7]Molecular Biophysics and Integrated Bioimaging Division, Lawrence Berkeley National Laboratory; Berkeley, CA, USA.

[8]Department of Chemistry, University of California, Berkeley; Berkeley, CA, USA.

*Corresponding author: doudna@berkeley.edu

[†]These authors contributed equally.

Supplementary table 1. Sequence information of CPPs used in this study.

CPP classes	CPP name	Copy #	Amino acid sequence
**Nuclear localization signals**	SV40 NLS	2X	PKKKRKV-GGS-PKKKRKV
	c-myc NLS	3X	PAAKRVKLD-GGS-PAAKRVKLD-GGS-PAAKRVKLD
	KP	2X	MAPTKRKGSCPGAAPNKKP-GGS-MAPTKRKGSCPGAAPNKKP
**Viral-derived peptides**	VP22	1X	NAATATRGRSAASRPTQRPRAPARSASRPRRP
	CA-Tat	1X-1x	KWKLFKKY-GRKKRRQRRRPPQ-GGS-GRKKRRQRRRPPQ
	CA-cTat	1X-1x	KWKLFKKY-GRKKRRQRRRPPQ-GGS-CYGRKKRRQRRRCGPKR
	HSV-gC	2X	GSRVQIRCRFRNSTR-GGS-GSRVQIRCRFRNSTR
	TD2.2	2X	SYWYRIVLSRTGRNGRLRVGRERPVLGESP-GGS-SYWYRIVLSRTGRNGRLRVGRERPVLGESP
**Cell-permeable miniature proteins**	ZF5.3	1X	MYSCNVCGKAFVLSRHLNRHLRVHRRAT
	YY2[5R1]	1X	APPLPPRNRGEDASPRELRRYYRSLRHYLNLVTRQRY
	aPP[6R1]	1X	GPSQPTYPGDDAPVRDLRRFYRDLRRYLNVVTRHRY
**Peptides from signaling proteins**	Penetratin	2X	RQIKIWFQNRRMKWKK-GGS-RQIKIWFQNRRMKWKK
	PenMax	2X	KWFKIQMQIRRWKNKR-GGS-KWFKIQMQIRRWKNKR
	EB1	2X	LIRLWSHLIHIWFQNRRLKWKKK-GGS-LIRLWSHLIHIWFQNRRLKWKKK
	DK17	2X	DRQIKIWFQNRRMKWKK-GGS-DRQIKIWFQNRRMKWKK
	TP10	2X	AGYLLGKINLKALAALAKKIL-GGS-AGYLLGKINLKALAALAKKIL
	M918	2X	MVTVLFRRLRIRRACGPPRVRV-GGS-MVTVLFRRLRIRRACGPPRVRV
**Peptides from other functional proteins**	A22p	2X	HTPGNSNKWKHLQENKKGRPRR-GGS-HTPGNSNKWKHLQENKKGRPRR
	HBP	2X	GKRKKKGKGLGKKRDPCLRKYK-GGS-GKRKKKGKGLGKKRDPCLRKYK
	hLF	2X	KCFQWQRNMRKVRGPPVSCIKR-GGS-KCFQWQRNMRKVRGPPVSCIKR
	BH	2X	AEATRLKRTARRRYTRRKNRIWYLQ-GGS-AEATRLKRTARRRYTRRKNRIWYLQ
	LALF	2X	HYRIKPTFRRLKWKYKGKFW-GGS-HYRIKPTFRRLKWKYKGKFW
	RT5.3	1X	RQIKIWFQNRRMKWKKAKLNAEKLKDFKIRLQY FARGLQVYIRQLRLALQGKT
	Arf22	2X	MVRRFLVTLRIRRACGPPRVRV-GGS-MVRRFLVTLRIRRACGPPRVRV
**Antimicrobial peptides**	Bac7	2X	RRIRPRPPRLPRPRPRPLPFPRPG-GGS-RRIRPRPPRLPRPRPRPLPFPRPG
	Crotamine	1X	YKQCHKKGGHCFPKEKICLPPSSDFGKMDCRWRWKCCKKGSG
	Buforin-II	2X	TRSSRAGLQFPVGRVHRLLRK-GGS-TRSSRAGLQFPVGRVHRLLRK
**Membrane-active peptides**	Pep-1	2X	KETWWETWWTEWSQPKKKRKV-GGS-KETWWETWWTEWSQPKKKRKV
	MPG	2X	GALFLGFLGAAGSTMGAWSQPKSKRKV-GGS-GALFLGFLGAAGSTMGAWSQPKSKRKV
	L17E	2X	IWLTALKFLGKHAAKHEAKQQLSKL-GGS-IWLTALKFLGKHAAKHEAKQQLSKL
	ATRAM	1X	GLAGLAGLLGLEGLLGLPLGLLEGLWLGLELEGN
	HA2E5-TAT	2X	GLFEAIAEFIENGWEGLIEGWYG-GGS-GLFEAIAEFIENGWEGLIEGWYG
	Mel-P1	1X	GIGAVLKVLATGLPALISWIKRKRQQ
	MPG	2X	GALFLGFLGAAGSTMGAWSQPKSKRKV-GGS-GALFLGFLGAAGSTMGAWSQPKSKRKV
	C6M1	2X	RLWRLLWRLWRRLWRLLR-GGS-RLWRLLWRLWRRLWRLLR

Supplementary table 2. Primer sequences for NGS amplicon sequencing. All primers for Illumina MiSeq were ordered with (5'-GCTCTTCCGATCT-3') at the 5' end for library preparation and indexing.

Primer name	Sequence (5'->3')	Application
Th-fwd	AATACCACAGCCTCCAATGGGTTCCCA	TH target (short read Illumina MiSeq)
Th-rev	TGATTAGCATGTTAGTCCTCCACTCCT	TH target (short read Illumina MiSeq)
mGluR5-ext-fwd	ACTAGGAAGCTCTCTTTCACAGGTATGTCTGC	mGluR5 target (1.1kb amplicon)
mGluR5-ext-rev	GAAAGTATCACATACCTTCTGTGTGCACAGCT	mGluR5 target (1.1kb amplicon)
mGluR5-fwd	TACTGCCTAATTCCTTAATGCACCAC	mGluR5 target (short read Illumina MiSeq)
mGluR5-rev	AACTTTGTCCACAGTTGGTTGGTGGTG	mGluR5 target (short read Illumina MiSeq)
BS-272-fwd	GCTCCTGGGCAACGTGCTGGTTATTG	tdTomato target (long read PacBio)
BS-273-rev	TTGATGACCTCCTCGCCCTTGCTCAC	tdTomato target (long read PacBio)
mGlutR5-fwd	GCTGTGAGATAAGAGATTCCTGC	mGluR5 target (qPCR)
mGlutR5-rev	ACTCCCACTATGGGTTTCTTGG	mGluR5 target (qPCR)
TH-fwd	GCCGTCTCAGAGCAGGATAC	TH target (qPCR)
TH-rev	TCCTCGAATACCACAGCCTC	TH target (qPCR)
mGAPDH-fwd	AACTTTGGCATTGTGGAAGG	mGAPDH target (qPCR)
mGAPDH-rev	CACATTGGGGGTAGGAACAC	mGAPDH target (qPCR)

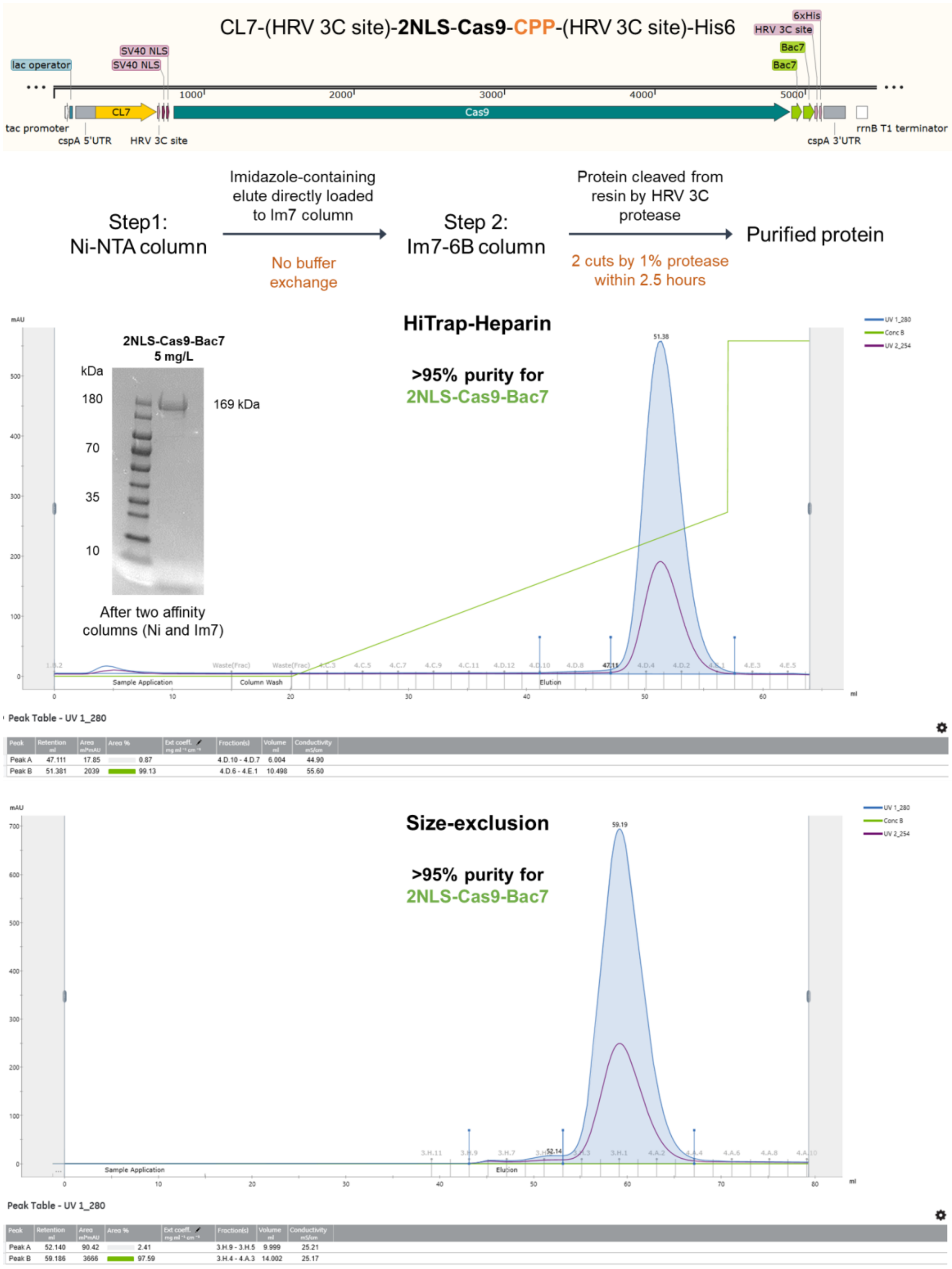

HiTrap-Heparin — Peak Table - UV 1_280

Peak	Retention ml	Area ml*mAU	Area %	Ext coeff. mg ml⁻¹ cm⁻¹	Fraction(s)	Volume ml	Conductivity mS/cm
Peak A	47.111	17.85	0.87		4.D.10 - 4.D.7	6.004	44.90
Peak B	51.381	2039	99.13		4.D.6 - 4.E.1	10.498	55.60

Size-exclusion — Peak Table - UV 1_280

Peak	Retention ml	Area ml*mAU	Area %	Ext coeff. mg ml⁻¹ cm⁻¹	Fraction(s)	Volume ml	Conductivity mS/cm
Peak A	52.140	90.42	2.41		3.H.9 - 3.H.5	9.999	25.21
Peak B	59.186	3666	97.59		3.H.4 - 4.A.3	14.002	25.17

Supplementary figure 1. Example of double-tagged purification. Tag-purified protein (2NLS-Cas9-Bac7) was further loaded to HiTrap-heparin and size-exclusion columns for elution, and <5% impurity was observed.

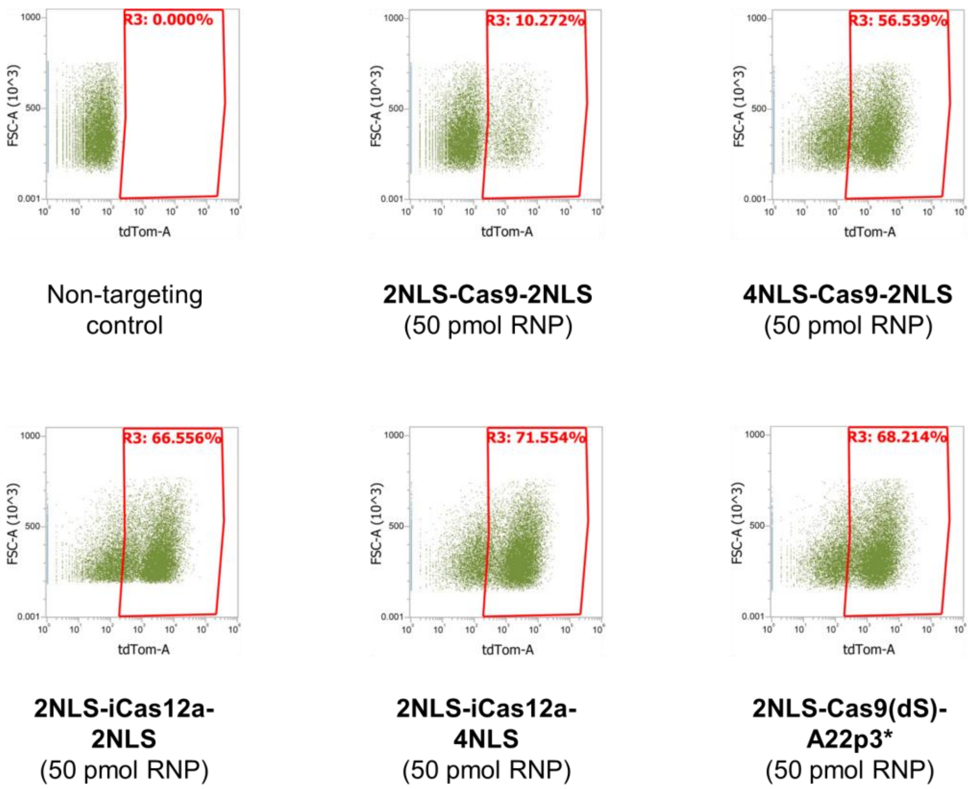

Supplementary figure 2. Flow analysis examples of genome editing activities with Ai9 tdTomato NPCs based on direct delivery of RNPs.

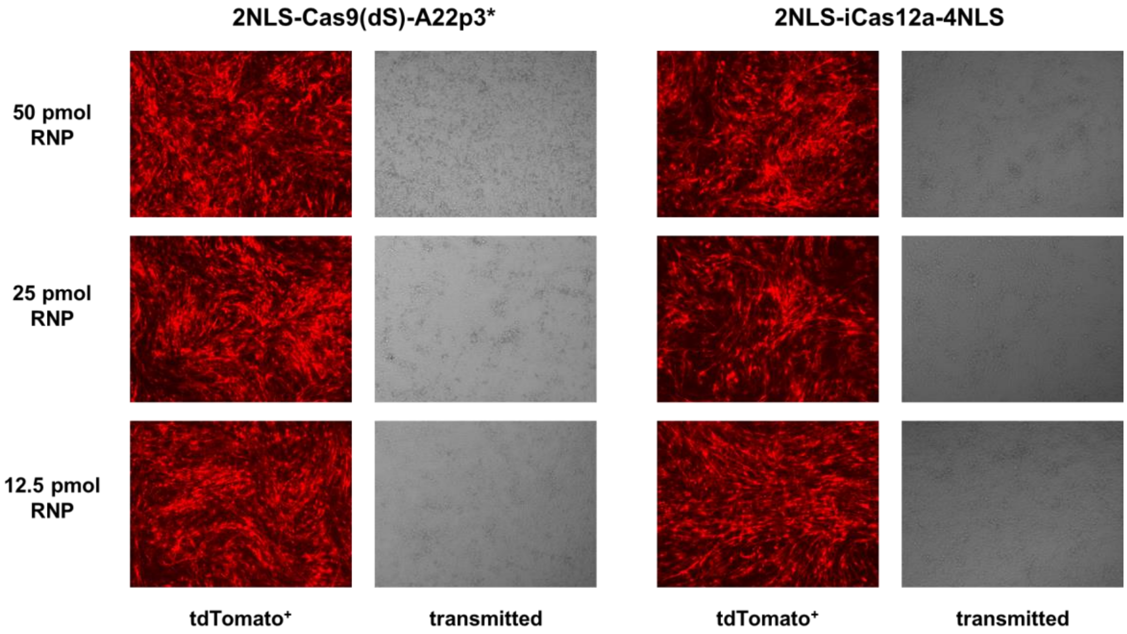

Supplementary figure 3. Imaging examples of genome editing activities with Ai9 tdTomato NPCs.

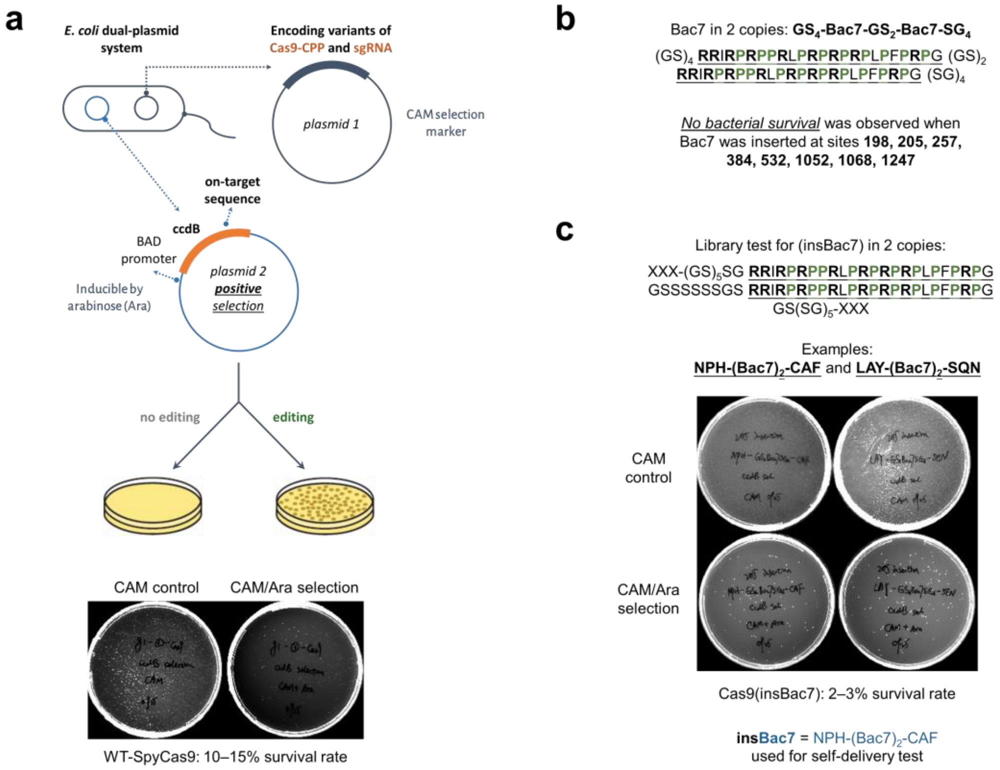

Supplementary figure 4. Engineering Cas9 with Bac7 peptide inserted into protein backbone. **a**. Bacterial selection assay used to assess the endonuclease activity of Cas9 or engineered Cas9 mutants with CPP inserted into the protein backbone. **b**. Evaluation of designed Cas9 mutants with Bac7 peptide inserted at different sites on Cas9. **c**. Library test for Cas9 mutants with redesigned Bac7 bearing longer linkers and three randomized flanking amino acids (XXX) on the two ends inserted at site 205, and evaluation of selected mutants in the bacterial assay.

a

CPP	#	Construct	Changes to Cas9	N-terminal sequence	C-terminal sequence
**Bac7** (RRIRPRPPRLPR PRPRPLPFPRPG)	1	2NLS-Cas9-Bac7	No change	S1-(SV40 NLS)$_2$-S2	GS-(Bac7)$_2$-S3
	2	Bac7-Cas9-2NLS	No change	S1-(Bac7)$_2$-S2	GS-(SV40 NLS)$_2$-S3
	3	c-myc NLS-Cas9-Bac7	No change	S1-(c-myc NLS)$_3$-S2	GS-(Bac7)$_2$-S3
	4	Bac7-Cas9-c-myc NLS	No change	S1-(Bac7)$_2$-S2	GS-(c-myc NLS)$_3$-S3
	5	2NLS-Cas9-Bac7*	No change	S1-(SV40 NLS)$_2$-S2	GS-(Bac7)$_2$
	6	2NLS-Cas9(insBac7)-2NLS	(Bac7)$_2$ at S205	S1-(SV40 NLS)$_2$-S2	GS-(SV40 NLS)$_2$-S3
**A22p** (HTPGNSNKWKH LQENKKGRPRR)	7	2NLS-Cas9-A22p	No change	S1-(SV40 NLS)$_2$-S2	GS-(A22p)$_2$-S3
	8	A22p-Cas9-2NLS	No change	S1-(A22p)$_2$-S2	GS-(SV40 NLS)$_2$-S3
	9	c-myc NLS-Cas9-A22p	No change	S1-(c-myc NLS)$_3$-S2	GS-(A22p)$_2$-S3
	10	A22p-Cas9-c-myc NLS	No change	S1-(A22p)$_2$-S2	GS-(c-myc NLS)$_3$-S3
	11	2NLS-Cas9-A22p*	No change	S1-(SV40 NLS)$_2$-S2	GS-(A22p)$_2$
	12	2NLS-Cas9-A22p3	No change	S1-(SV40 NLS)$_2$-S2	GS-(A22p)$_3$-S3
	13	2NLS-Cas9-A22p3*	No change	S1-(SV40 NLS)$_2$-S2	GS-(A22p)$_3$
	14	2NLS-Cas9(dS)-A22p3	C80S, C574S	S1-(SV40 NLS)$_2$-S2	GS-(A22p)$_3$-S3
	15	2NLS-Cas9(dS)-A22p3*	C80S, C574S	S1-(SV40 NLS)$_2$-S2	GS-(A22p)$_3$
**VP22** (NAATATRGRSAASRPTQRPRA PARSASRPRRP)	16	2NLS-Cas9-VP22	No change	S1-(SV40 NLS)$_2$-S2	GS-VP22-S3
	17	VP22-Cas9-2NLS	No change	S1-VP22-S2	GS-(SV40 NLS)$_2$-S3
	18	c-myc NLS-Cas9-VP22	No change	S1-(c-myc NLS)$_3$-S2	GS-VP22-S3
**KP** (MAPTKRKGSCPGAAPNKKP)	19	2NLS-Cas9-KP	No change	S1-(SV40 NLS)$_2$-S2	GS-(KP)$_2$-S3
	20	KP-Cas9-2NLS	No change	S1-(KP)$_2$-S2	GS-(SV40 NLS)$_2$-S3
**CA-Tat** (See Table S1)	21	2NLS-Cas9-CA-Tat	No change	S1-(SV40 NLS)$_2$-S2	GS-CA-(Tat)$_2$-S3
**CA-cTat** (See Table S1)	22	2NLS-Cas9-CA-cTat	No change	S1-(SV40 NLS)$_2$-S2	GS-CA-Tat-cTat-S3

S1: GPNAT; S2: GIHGVPAAT; S3: SLEVLFQ. GGS as the linker for between copies of CPPs at *N*- and *C*-termini.

b

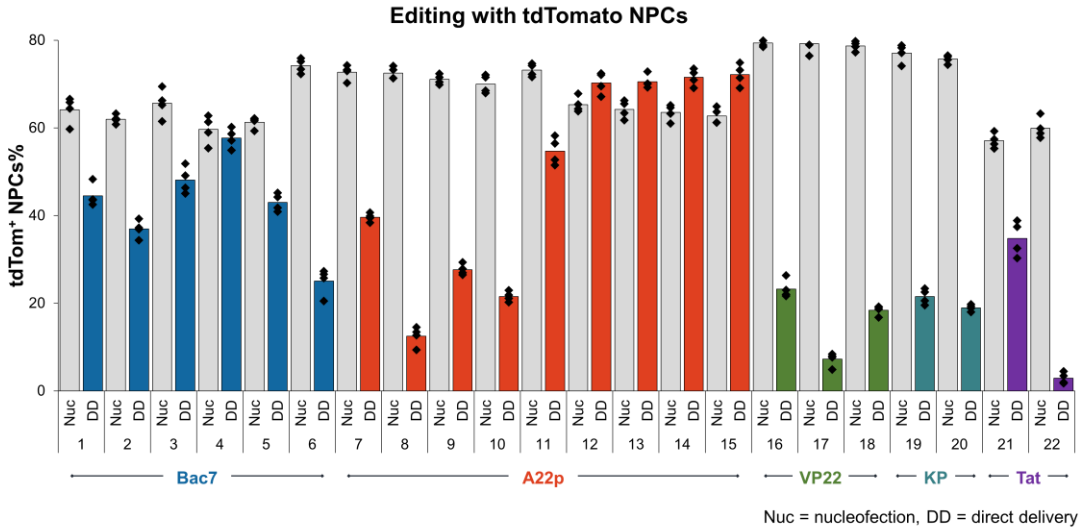

Supplementary figure 5. Engineering Cas9-CPP constructs for self-delivery to NPCs. **a.** Table of Cas9-CPP constructs. **b**. Evaluation of Cas9-CPP constructs for gene editing activities using Ai9 tdTom NPCs based on nucleofection and self-delivery of 100 pmol RNPs. n = 4 for each group, mean ± s.e.m. * cTat stands for cyclic Tat (peptide cyclization enabled by Cys disulfide ligation).

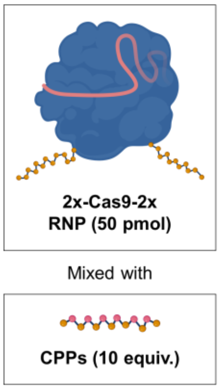

CPP name	Amino acid sequence
Bac7	RRIRPRPPRLPRPRPRPLPFPRPG
EB1	LIRLWSHLIHIWFQNRRLKWKKK
A22p	HTPGNSNKWKHLQENKKGRPRR
LALF	HYRIKPTFRRLKWKYKGKFW
PenMax	KWFKIQMQIRRWKNKR
KP	MAPTKRKGSCPGAAPNKKP
VP22	NAATATRGRSAASRPTQRPRAPARSASRPRRP

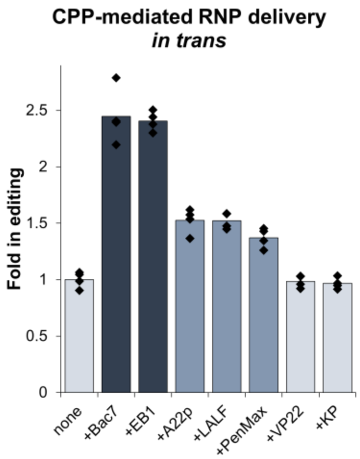

Supplementary figure 6. Peptide-assisted delivery of 2x-Cas9-2x RNP for genome editing in NPCs. n = 4 for each group, mean ± s.e.m. Images used in this figure were created with biorender.com.

Protein	M.W.	RNP M.W.	Net charge of protein	Charge of sgRNA	Net charge of RNP
peptide-free Cas9	159 kDa	191 kDa	20(+)	100(–)	80(–)
2NLS-Cas9(dS)-A22p3*	170 KDa	202 kDa	48(+)	100(–)	52(–)

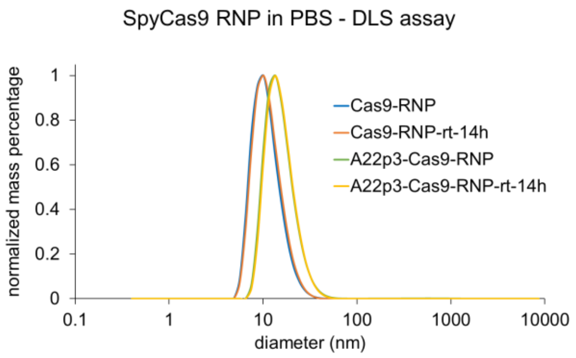

Cas9 RNP	RNP M.W.	Aver. RNP Size by DLS	RNP Zeta Potential
peptide-free Cas9/sgRNA	191 kDa	11.4 nm	–4.01 ± 0.44 mV
2NLS-Cas9(dS)-A22p3*/sgRNA	202 kDa	15.8 nm	–2.61 ± 0.46 mV

Supplementary figure 7. Characterization of Cas9-A22p3* RNP in comparison with RNP based on non-tagged Cas9. rt-14h: incubation at room temperature for 14 hours. No substantial change in particle size after incubating RNPs at room temperature overnight for 14 hours. The shifted size distribution of A22p3*-fused RNP in comparison to peptide-free RNP may indicate some oligomeric states associated with A22p3*-fused RNP through self-assembly.

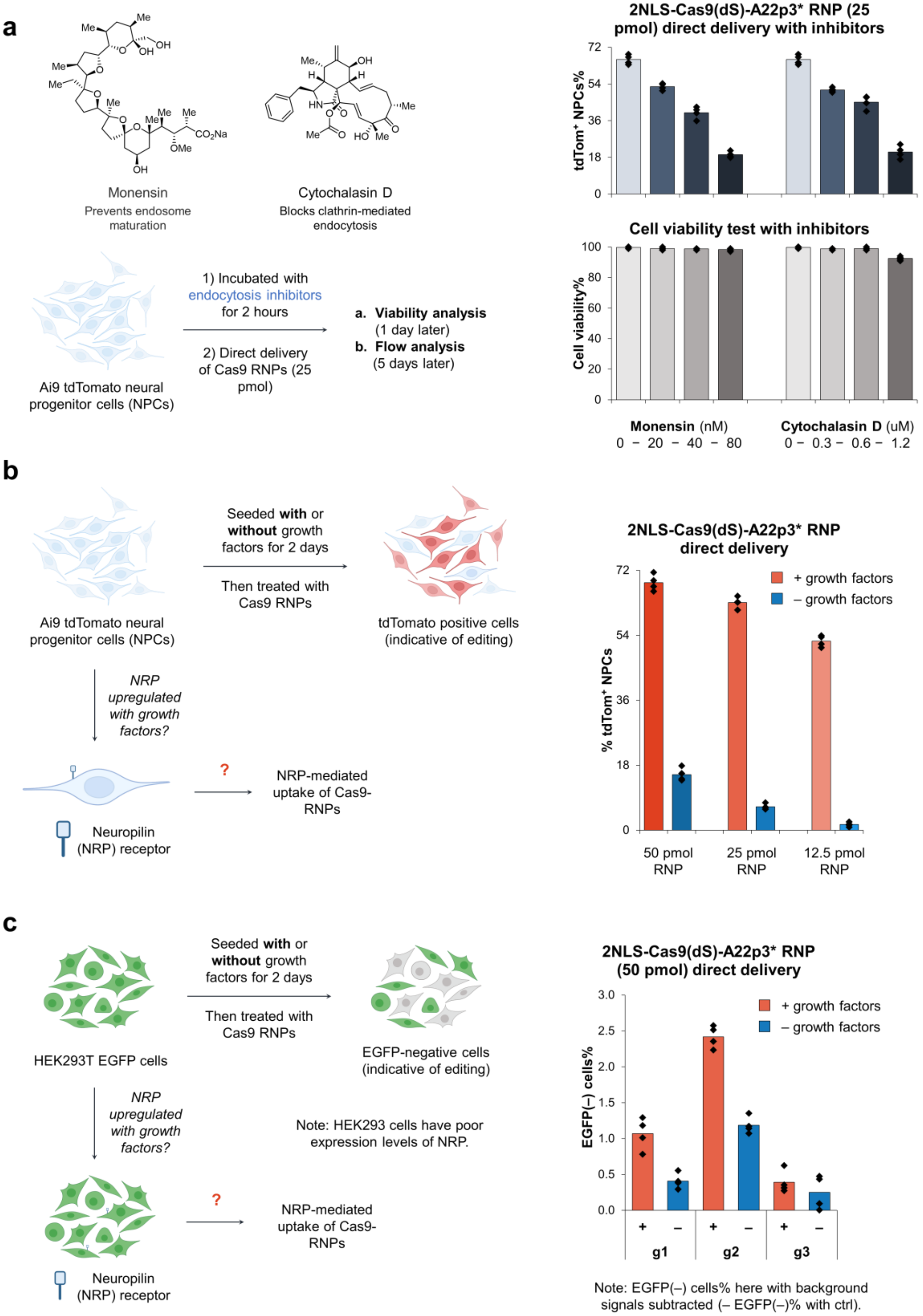

Supplementary figure 8. a. Evaluation of Cas9-A22p3* construct for self-delivery abilities in the presence of endocytosis inhibitors of different doses. **b**. Evaluation of Cas9-A22p3* construct for self-delivery abilities with or without growth factors in NPCs, and hypothesis on the functions of

growth factors. **c**. Effect of growth factors on the self-delivery abilities of Cas9-A22p3* RNPs in HEK293T cells. n = 4 for each group, mean ± s.e.m. Images used in this figure were created with biorender.com.

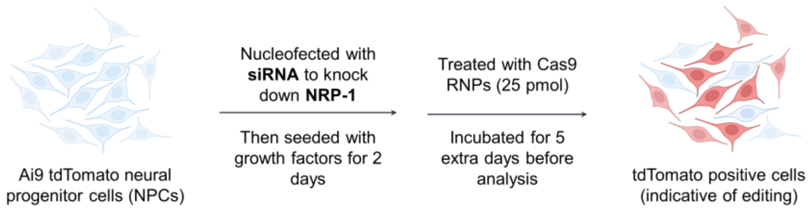

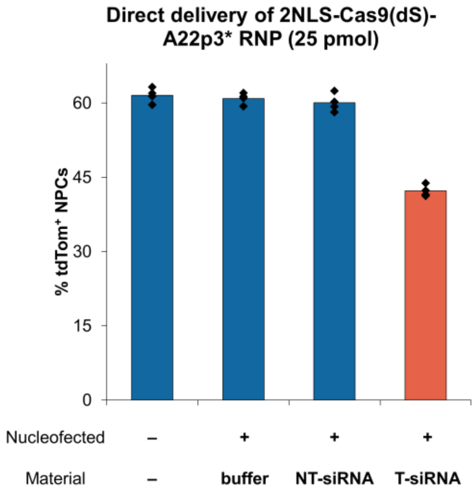

Supplementary figure 9. Effect of NRP-1 knockdown on the efficiency of direct Cas9 RNP delivery. n = 4 for each group, mean ± s.e.m. NT: non-targeting; T: targeting. NPCs with NRP-1 knocked down by siRNA showed reduced efficiency for direct RNP delivery, which indicates that NRP-1 may be involved in the cellular uptake of RNPs. Meanwhile, this finding also implies that other uncharacterized cell-surface receptors may also play a role in RNP uptake. Images used in this figure were created with biorender.com.

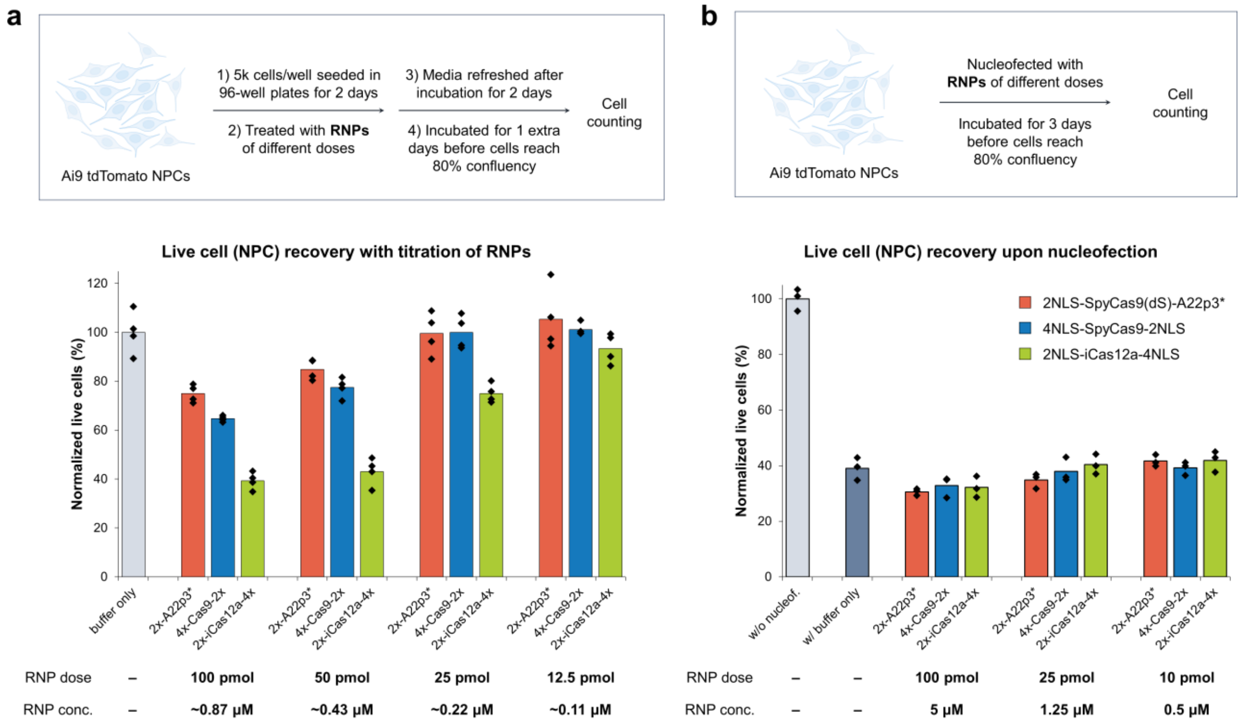

Supplementary figure 10. Cell viability comparison based on RNP delivery by direct treatment or nucleofection. **a.** Cell viability comparison based on the direct delivery of different RNPs at different doses. **b.** Cell viability comparison based on nucleofection of different RNPs at different doses. n = 4 for each group in panel **a** and n =3 for each group in panel **b**, mean ± s.e.m. Cell-permeable Cas9 RNPs showed minimal effect on NPC growth at low doses (25 or 12.5 pmol, 0.22 or 0.11 μM, respectively); iCas12a RNP had a more significant effect on NPC growth. Nucleofection showed high toxicity to NPCs, generally leading to <40% live cell recovery in the presence or absence of RNPs. Images used in this figure were created with biorender.com.

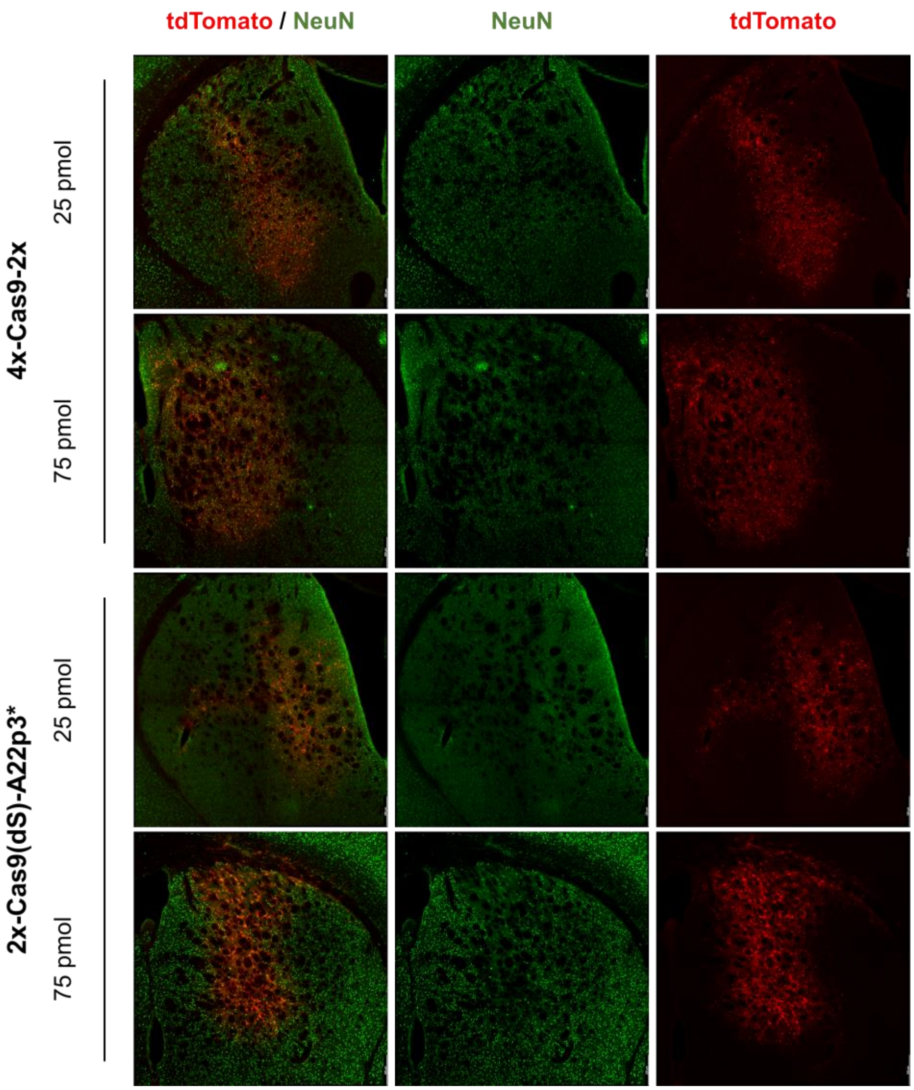

Supplementary figure 11. Examples of brain slice imaging with signals showing the expression of tdTomato (red) and NeuN (green).

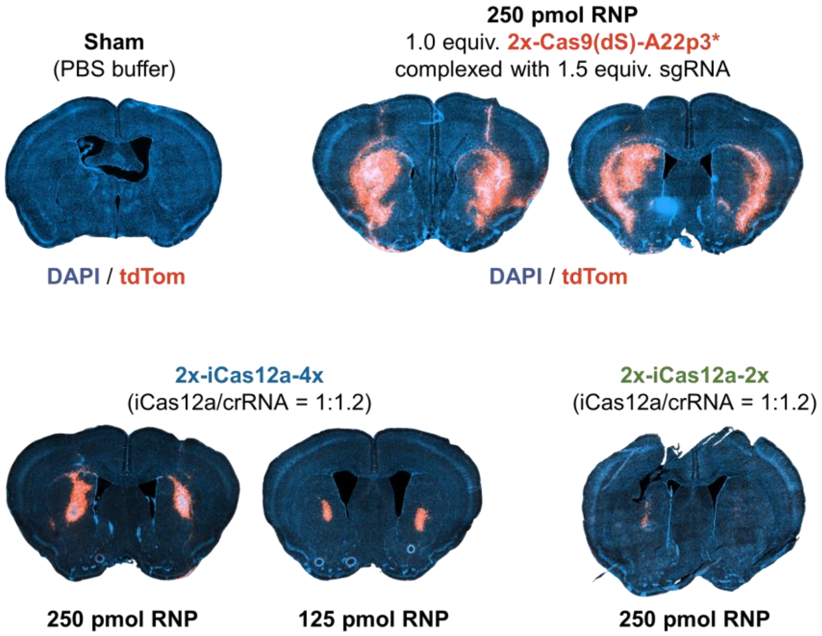

Supplementary figure 12. Genome editing in the brain of Ai9 mice with different RNP constructs.

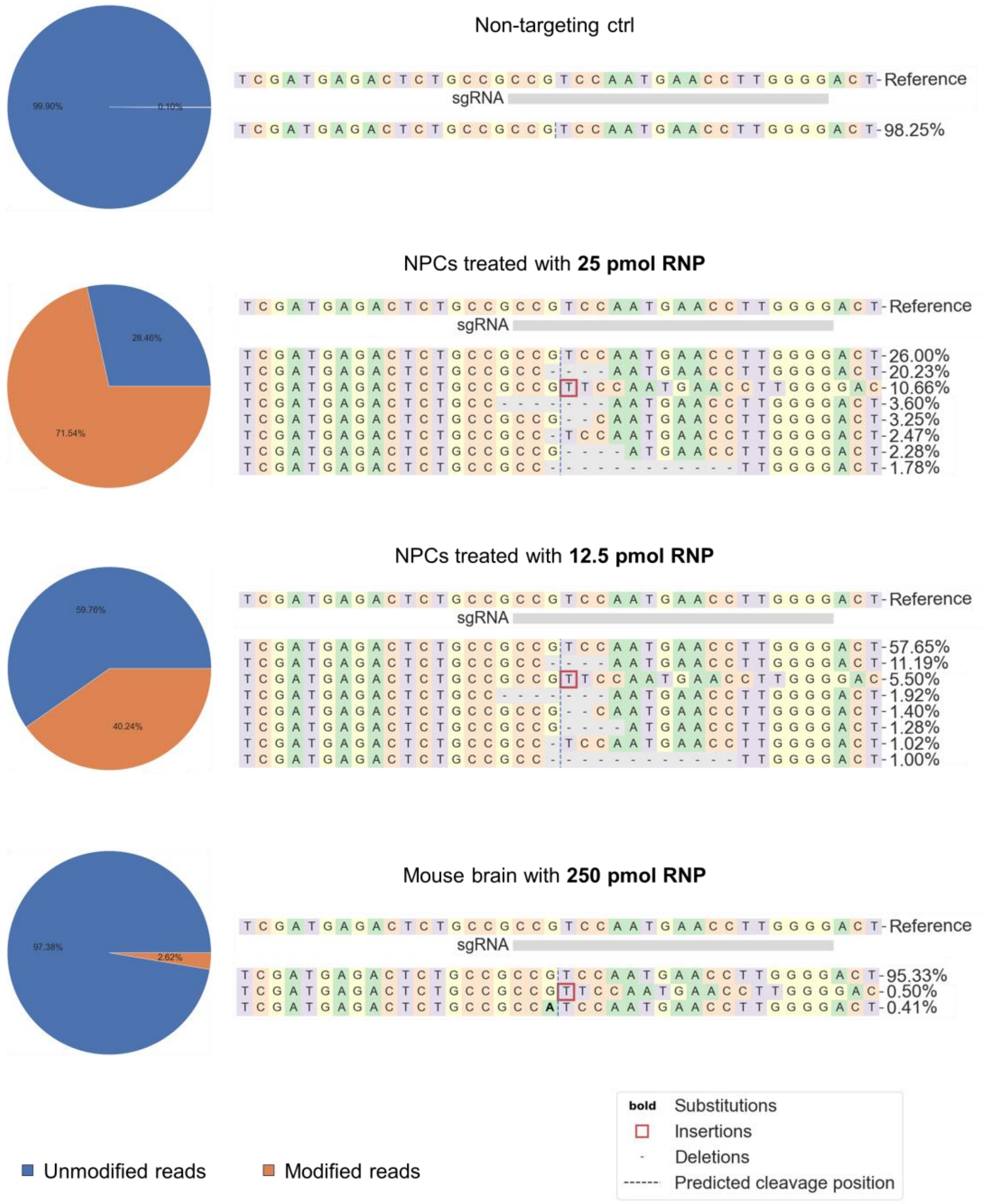

Supplementary figure 13. NGS analysis to quantify genome editing levels targeting tyrosine hydroxylase (TH).

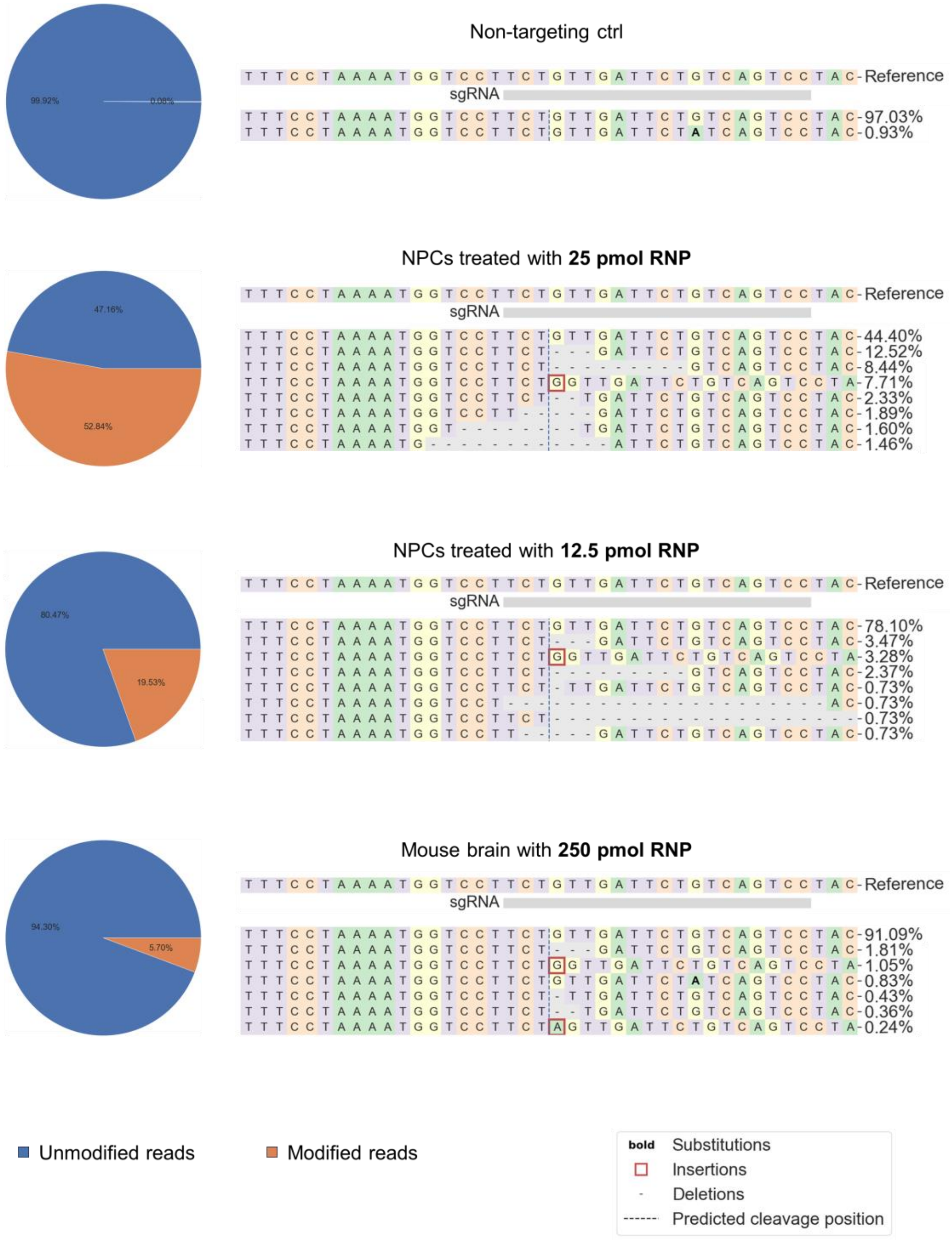

Supplementary figure 14. NGS analysis to quantify genome editing levels targeting metabotropic glutamate receptor 5 (mGluR5).

Protein sequences:

SpyCas9, iCas12a, SV40 NLS, A22p peptide, CL7 tag, His tag, Linkers

HRV protease recognition site, TEV protease recognition site (↓: protease cleavage position)

Expression construct of 4x-Cas9-2x:

MKSSHHHHHHENLYFQ↓SNENLYFQ↓SNATPKKRKVGGSPKKRKVGGSPKKRKVGGSPKKRKVGIHGVPAA
TMDKKYSIGLDIGTNSVGWAVITDEYKVPSKKFKVLGNTDRHSIKKNLIGALLFDSGETAEATRLKRTAR
RRYTRRKNRICYLQEIFSNEMAKVDDSFFHRLEESFLVEEDKKHERHPIFGNIVDEVAYHEKYPTIYHLR
KKLVDSTDKADLRLIYLALAHMIKFRGHFLIEGDLNPDNSDVDKLFIQLVQTYNQLFEENPINASGVDAK
AILSARLSKSRRLENLIAQLPGEKKNGLFGNLIALSLGLTPNFKSNFDLAEDAKLQLSKDTYDDDLDNLL
AQIGDQYADLFLAAKNLSDAILLSDILRVNTEITKAPLSASMIKRYDEHHQDLTLLKALVRQQLPEKYKE
IFFDQSKNGYAGYIDGGASQEEFYKFIKPILEKMDGTEELLVKLNREDLLRKQRTFDNGSIPHQIHLGEL
HAILRRQEDFYPFLKDNREKIEKILTFRIPYYVGPLARGNSRFAWMTRKSEETITPWNFEEVVDKGASAQ
SFIERMTNFDKNLPNEKVLPKHSLLYEYFTVYNELTKVKYVTEGMRKPAFLSGEQKKAIVDLLFKTNRKV
TVKQLKEDYFKKIECFDSVEISGVEDRFNASLGTYHDLLKIIKDKDFLDNEENEDILEDIVLTLTLFEDR
EMIEERLKTYAHLFDDKVMKQLKRRRYTGWGRLSRKLINGIRDKQSGKTILDFLKSDGFANRNFMQLIHD
DSLTFKEDIQKAQVSGQGDSLHEHIANLAGSPAIKKGILQTVKVVDELVKVMGRHKPENIVIEMARENQT
TQKGQKNSRERMKRIEEGIKELGSQILKEHPVENTQLQNEKLYLYYLQNGRDMYVDQELDINRLSDYDVD
HIVPQSFLKDDSIDNKVLTRSDKNRGKSDNVPSEEVVKKMKNYWRQLLNAKLITQRKFDNLTKAERGGLS
ELDKAGFIKRQLVETRQITKHVAQILDSRMNTKYDENDKLIREVKVITLKSKLVSDFRKDFQFYKVREIN
NYHHAHDAYLNAVVGTALIKKYPKLESEFVYGDYKVYDVRKMIAKSEQEIGKATAKYFFYSNIMNFFKTE
ITLANGEIRKRPLIETNGETGEIVWDKGRDFATVRKVLSMPQVNIVKKTEVQTGGFSKESILPKRNSDKL
IARKKDWDPKKYGGFDSPTVAYSVLVVAKVEKGKSKKLKSVKELLGITIMERSSFEKNPIDFLEAKGYKE
VKKDLIIKLPKYSLFELENGRKRMLASAGELQKGNELALPSKYVNFLYLASHYEKLKGSPEDNEQKQLFV
EQHKHYLDEIIEQISEFSKRVILADANLDKVLSAYNKHRDKPIREQAENIIHLFTLTNLGAPAAFKYFDT
TIDRKRYTSTKEVLDATLIHQSITGLYETRIDLSQLGGDGSPKKKRKVEDPKKKRKV

Expression constructs of 2x-Cas9-2x (2 versions):

2x-Cas9-2x used for Figure 1:

MKSSHHHHHHENLYFQ↓SNENLYFQ↓SNATPKKRKVGGSPKKRKVGIHGVPAATMDKKYSIGLDIGTNSVG
WAVITDEYKVPSKKFKVLGNTDRHSIKKNLIGALLFDSGETAEATRLKRTARRRYTRRKNRICYLQEIFS
NEMAKVDDSFFHRLEESFLVEEDKKHERHPIFGNIVDEVAYHEKYPTIYHLRKKLVDSTDKADLRLIYLA
LAHMIKFRGHFLIEGDLNPDNSDVDKLFIQLVQTYNQLFEENPINASGVDAKAILSARLSKSRRLENLIA
QLPGEKKNGLFGNLIALSLGLTPNFKSNFDLAEDAKLQLSKDTYDDDLDNLLAQIGDQYADLFLAAKNLS
DAILLSDILRVNTEITKAPLSASMIKRYDEHHQDLTLLKALVRQQLPEKYKEIFFDQSKNGYAGYIDGGA
SQEEFYKFIKPILEKMDGTEELLVKLNREDLLRKQRTFDNGSIPHQIHLGELHAILRRQEDFYPFLKDNR
EKIEKILTFRIPYYVGPLARGNSRFAWMTRKSEETITPWNFEEVVDKGASAQSFIERMTNFDKNLPNEKV
LPKHSLLYEYFTVYNELTKVKYVTEGMRKPAFLSGEQKKAIVDLLFKTNRKVTVKQLKEDYFKKIECFDS
VEISGVEDRFNASLGTYHDLLKIIKDKDFLDNEENEDILEDIVLTLTLFEDREMIEERLKTYAHLFDDKV
MKQLKRRRYTGWGRLSRKLINGIRDKQSGKTILDFLKSDGFANRNFMQLIHDDSLTFKEDIQKAQVSGQG
DSLHEHIANLAGSPAIKKGILQTVKVVDELVKVMGRHKPENIVIEMARENQTTQKGQKNSRERMKRIEEG
IKELGSQILKEHPVENTQLQNEKLYLYYLQNGRDMYVDQELDINRLSDYDVDHIVPQSFLKDDSIDNKVL
TRSDKNRGKSDNVPSEEVVKKMKNYWRQLLNAKLITQRKFDNLTKAERGGLSELDKAGFIKRQLVETRQI
TKHVAQILDSRMNTKYDENDKLIREVKVITLKSKLVSDFRKDFQFYKVREINNYHHAHDAYLNAVVGTAL
IKKYPKLESEFVYGDYKVYDVRKMIAKSEQEIGKATAKYFFYSNIMNFFKTEITLANGEIRKRPLIETNG
ETGEIVWDKGRDFATVRKVLSMPQVNIVKKTEVQTGGFSKESILPKRNSDKLIARKKDWDPKKYGGFDSP
TVAYSVLVVAKVEKGKSKKLKSVKELLGITIMERSSFEKNPIDFLEAKGYKEVKKDLIIKLPKYSLFELE
NGRKRMLASAGELQKGNELALPSKYVNFLYLASHYEKLKGSPEDNEQKQLFVEQHKHYLDEIIEQISEFS
KRVILADANLDKVLSAYNKHRDKPIREQAENIIHLFTLTNLGAPAAFKYFDTTIDRKRYTSTKEVLDATL
IHQSITGLYETRIDLSQLGGDGSPKKKRKVEDPKKKRKV

2x-Cas9-2x used for Figure 2 &3:

MSKSNEPGKATGEGKPVNNKWLNNAGKDLGSPVPDRIANKLRDKEFESFDDFRETFWEEVSKDPELSKQF
SRNNNDRMKVGKAPKTRTQDVSGKRTSFELNHQKPIEQNGGVYDMDNISVVTPKRNIDIEGGGGGSLEVL
FQ↓GPNATPKKRKVGGSPKKRKVGIHGVPAATMDKKYSIGLDIGTNSVGWAVITDEYKVPSKKFKVLGNTD
RHSIKKNLIGALLFDSGETAEATRLKRTARRRYTRRKNRICYLQEIFSNEMAKVDDSFFHRLEESFLVEE
DKKHERHPIFGNIVDEVAYHEKYPTIYHLRKKLVDSTDKADLRLIYLALAHMIKFRGHFLIEGDLNPDNS
DVDKLFIQLVQTYNQLFEENPINASGVDAKAILSARLSKSRRLENLIAQLPGEKKNGLFGNLIALSLGLT
PNFKSNFDLAEDAKLQLSKDTYDDDLDNLLAQIGDQYADLFLAAKNLSDAILLSDILRVNTEITKAPLSA
SMIKRYDEHHQDLTLLKALVRQQLPEKYKEIFFDQSKNGYAGYIDGGASQEEFYKFIKPILEKMDGTEEL
LVKLNREDLLRKQRTFDNGSIPHQIHLGELHAILRRQEDFYPFLKDNREKIEKILTFRIPYYVGPLARGN
SRFAWMTRKSEETITPWNFEEVVDKGASAQSFIERMTNFDKNLPNEKVLPKHSLLYEYFTVYNELTKVKY
VTEGMRKPAFLSGEQKKAIVDLLFKTNRKVTVKQLKEDYFKKIECFDSVEISGVEDRFNASLGTYHDLLK
IIKDKDFLDNEENEDILEDIVLTLTLFEDREMIEERLKTYAHLFDDKVMKQLKRRRYTGWGRLSRKLING
IRDKQSGKTILDFLKSDGFANRNFMQLIHDDSLTFKEDIQKAQVSGQGDSLHEHIANLAGSPAIKKGILQ
TVKVVDELVKVMGRHKPENIVIEMARENQTTQKGQKNSRERMKRIEEGIKELGSQILKEHPVENTQLQNE
KLYLYYLQNGRDMYVDQELDINRLSDYDVDHIVPQSFLKDDSIDNKVLTRSDKNRGKSDNVPSEEVVKKM
KNYWRQLLNAKLITQRKFDNLTKAERGGLSELDKAGFIKRQLVETRQITKHVAQILDSRMNTKYDENDKL
IREVKVITLKSKLVSDFRKDFQFYKVREINNYHHAHDAYLNAVVGTALIKKYPKLESEFVYGDYKVYDVR
KMIAKSEQEIGKATAKYFFYSNIMNFFKTEITLANGEIRKRPLIETNGETGEIVWDKGRDFATVRKVLSM
PQVNIVKKTEVQTGGFSKESILPKRNSDKLIARKKDWDPKKYGGFDSPTVAYSVLVVAKVEKGKSKKLKS
VKELLGITIMERSSFEKNPIDFLEAKGYKEVKKDLIIKLPKYSLFELENGRKRMLASAGELQKGNELALP
SKYVNFLYLASHYEKLKGSPEDNEQKQLFVEQHKHYLDEIIEQISEFSKRVILADANLDKVLSAYNKHRD
KPIREQAENIIHLFTLTNLGAPAAFKYFDTTIDRKRYTSTKEVLDATLIHQSITGLYETRIDLSQLGGDG
SPKKKRKVEDPKKKRKVSLEVLFQ↓GPGSHHHHHH

Expression construct of 2x-iCas12a-2x:

MKSSHHHHHHGSSKSNEPGKATGEGKPVNNKWLNNAGKDLGSPVPDRIANKLRDKEFESFDDFRETFWEE
VSKDPELSKQFSRNNNDRMKVGKAPKTRTQDVSGKRTSFELNHQKPIEQNGGVYDMDNISVVTPKRNIDI
EGGGGGSLEVLFQ↓GPNATPKKRKVGGSPKKRKVGIHGVPAATMSKLEKFTNCYSLSKTLRFKAIPVGKT
QENIDNKRLLVEDEKRAEDYKGVKKLLDRYYLSFINDVLHSIKLKNLNNYISLFRKKTRTEKENKELENL
EINLRKEIAKAFKGNEGYKSLFKKDIIETILPEFLDDKDEIALVNSFNGFTTAFTGFFDNRENMFSEEAK
STSIAFRCINENLTRYISNMDIFEKVDAIFDKHEVQEIKEKILNSDYDVEDFFEGEFFNFVLTQEGIDVY
NAIIGGFVTESGEKIKGLNEYINLYNQKTKQKLPKFKPLYKQVLSDRESLSFYGEGYTSDEEVLEVFRNT
LNKNSEIFSSIKKLEKLFKNFDEYSSAGIFVKNGPAISTISKDIFGEWNVIRDKWNAEYDDIHLKKKAVV
TEKYEDDRRKSFKKIGSFSLEQLQEYADADLSVVEKLKEIIIQKVDEIYKVYGSSEKLFDADFVLEKSLK
KNDAVVAIMKDLLDSVKSFENYIKAFFGEGKETNRDESFYGDFVLAYDILLKVDHIYDAIRNYVTQKPYS
KDKFKLYFQNPQFMGGWDKDKETDYRATILRYGSKYYLAIMDKKYAKCLQKIDKDDVNGNYEKINYKLLP
GPNKMLPKVFFSKKWMAYYNPSEDIQKIYKNGTFKKGDMFNLNDCHKLIDFFKDSISRYPKWSNAYDFNF
SETEKYKDIAGFYREVEEQGYKVSFESASKKEVDKLVEEGKLYMFQIYNKDFSDKSHGTPNLHTMYFKLL
FDENNHGQIRLSGGAELFMRRASLKKEELVVHPANSPIANKNPDNPKKTTTLSYDVYKDKRFSEDQYELH
IPIAINKCPKNIFKINTEVRVLLKHDDNPYVIGIDRGERNLLYIVVVDGKGNIVEQYSLNEIINNVNGIR
IKTDYHSLLDKKEKERFEARQNWTSIENIKELKAGYISQVVHKICELVEKYDAVIALEDLNSGFKNSRVK
VEKQVYQKFEKMLINKLNYMVDKKSNPYATGGALKGYQITNKFESFKSMSTQNGFIFYIPAWLTSKIDPS
TGFANLLKTKYTSIADSKKFISSFDRIMYVPEEDLFEFALDYKNFSRTDADYIKKWKLYSYGNRIRIFRN
PKKNNVFDWEEVCLTSAYKELFNKYGINYQLGDIRVLLCEQSDKAFYSSFMALMTLMLQMRNSITGRTDV
DFLISPVKNSDGIFYDSRNYEAQENAILPKNADANGAYNIARKVLWAIGQFKKAEDEKLDKVKIAIPNKE
WLEYAQTSVKHGGSPKKRKVGGSPKKRKV

Expression construct of 2x-iCas12a-4x:

MKSSHHHHHHGSSKSNEPGKATGEGKPVNNKWLNNAGKDLGSPVPDRIANKLRDKEFESFDDFRETFWEE

VSKDPELSKQFSRNNNDRMKVGKAPKTRTQDVSGKRTSFELNHQKPIEQNGGVYDMDNISVVTPKRNIDI
EGGGGGSLEVLFQ↓GPNATPKKRKVGGSPKKRKVGIHGVPAATMSKLEKFTNCYSLSKTLRFKAIPVGKT
QENIDNKRLLVEDEKRAEDYKGVKKLLDRYYLSFINDVLHSIKLKNLNNYISLFRKKTRTEKENKELENL
EINLRKEIAKAFKGNEGYKSLFKKDIIETILPEFLDDKDEIALVNSFNGFTTAFTGFFDNRENMFSEEAK
STSIAFRCINENLTRYISNMDIFEKVDAIFDKHEVQEIKEKILNSDYDVEDFFEGEFFNFVLTQEGIDVY
NAIIGGFVTESGEKIKGLNEYINLYNQKTKQKLPKFKPLYKQVLSDRESLSFYGEGYTSDEEVLEVFRNT
LNKNSEIFSSIKKLEKLFKNFDEYSSAGIFVKNGPAISTISKDIFGEWNVIRDKWNAEYDDIHLKKKAVV
TEKYEDDRRKSFKKIGSFSLEQLQEYADADLSVVEKLKEIIIQKVDEIYKVYGSSEKLFDADFVLEKSLK
KNDAVVAIMKDLLDSVKSFENYIKAFFGEGKETNRDESFYGDFVLAYDILLKVDHIYDAIRNYVTQKPYS
KDKFKLYFQNPQFMGGWDKDKETDYRATILRYGSKYYLAIMDKKYAKCLQKIDKDDVNGNYEKINYKLLP
GPNKMLPKVFFSKKWMAYYNPSEDIQKIYKNGTFKKGDMFNLNDCHKLIDFFKDSISRYPKWSNAYDFNF
SETEKYKDIAGFYREVEEQGYKVSFESASKKEVDKLVEEGKLYMFQIYNKDFSDKSHGTPNLHTMYFKLL
FDENNHGQIRLSGGAELFMRRASLKKEELVVHPANSPIANKNPDNPKKTTTLSYDVYKDKRFSEDQYELH
IPIAINKCPKNIFKINTEVRVLLKHDDNPYVIGIDRGERNLLYIVVVDGKGNIVEQYSLNEIINNVNGIR
IKTDYHSLLDKKEKERFEARQNWTSIENIKELKAGYISQVVHKICELVEKYDAVIALEDLNSGFKNSRVK
VEKQVYQKFEKMLINKLNYMVDKKSNPYATGGALKGYQITNKFESFKSMSTQNGFIFYIPAWLTSKIDPS
TGFANLLKTKYTSIADSKKFISSFDRIMYVPEEDLFEFALDYKNFSRTDADYIKKWKLYSYGNRIRIFRN
PKKNNVFDWEEVCLTSAYKELFNKYGINYQLGDIRVLLCEQSDKAFYSSFMALMTLMLQMRNSITGRTDV
DFLISPVKNSDGIFYDSRNYEAQENAILPKNADANGAYNIARKVLWAIGQFKKAEDEKLDKVKIAIPNKE
WLEYAQTSVKHGGSPKKRKVGGSPKKRKVGGSPKKRKVGGSPKKRKV

Expression construct of 2x-Cas9-A22p3:

MSKSNEPGKATGEGKPVNNKWLNNAGKDLGSPVPDRIANKLRDKEFESFDDFRETFWEEVSKDPELSKQF
SRNNNDRMKVGKAPKTRTQDVSGKRTSFELNHQKPIEQNGGVYDMDNISVVTPKRNIDIEGGGGGSLEVL
FQ↓GPNATPKKRKVGGSPKKRKVGIHGVPAATMDKKYSIGLDIGTNSVGWAVITDEYKVPSKKFKVLGNTD
RHSIKKNLIGALLFDSGETAEATRLKRTARRRYTRRKNRICYLQEIFSNEMAKVDDSFFHRLEESFLVEE
DKKHERHPIFGNIVDEVAYHEKYPTIYHLRKKLVDSTDKADLRLIYLALAHMIKFRGHFLIEGDLNPDNS
DVDKLFIQLVQTYNQLFEENPINASGVDAKAILSARLSKSRRLENLIAQLPGEKKNGLFGNLIALSLGLT
PNFKSNFDLAEDAKLQLSKDTYDDDLDNLLAQIGDQYADLFLAAKNLSDAILLSDILRVNTEITKAPLSA
SMIKRYDEHHQDLTLLKALVRQQLPEKYKEIFFDQSKNGYAGYIDGGASQEEFYKFIKPILEKMDGTEEL
LVKLNREDLLRKQRTFDNGSIPHQIHLGELHAILRRQEDFYPFLKDNREKIEKILTFRIPYYVGPLARGN
SRFAWMTRKSEETITPWNFEEVVDKGASAQSFIERMTNFDKNLPNEKVLPKHSLLYEYFTVYNELTKVKY
VTEGMRKPAFLSGEQKKAIVDLLFKTNRKVTVKQLKEDYFKKIECFDSVEISGVEDRFNASLGTYHDLLK
IIKDKDFLDNEENEDILEDIVLTLTLFEDREMIEERLKTYAHLFDDKVMKQLKRRRYTGWGRLSRKLING
IRDKQSGKTILDFLKSDGFANRNFMQLIHDDSLTFKEDIQKAQVSGQGDSLHEHIANLAGSPAIKKGILQ
TVKVVDELVKVMGRHKPENIVIEMARENQTTQKGQKNSRERMKRIEEGIKELGSQILKEHPVENTQLQNE
KLYLYYLQNGRDMYVDQELDINRLSDYDVDHIVPQSFLKDDSIDNKVLTRSDKNRGKSDNVPSEEVVKKM
KNYWRQLLNAKLITQRKFDNLTKAERGGLSELDKAGFIKRQLVETRQITKHVAQILDSRMNTKYDENDKL
IREVKVITLKSKLVSDFRKDFQFYKVREINNYHHAHDAYLNAVVGTALIKKYPKLESEFVYGDYKVYDVR
KMIAKSEQEIGKATAKYFFYSNIMNFFKTEITLANGEIRKRPLIETNGETGEIVWDKGRDFATVRKVLSM
PQVNIVKKTEVQTGGFSKESILPKRNSDKLIARKKDWDPKKYGGFDSPTVAYSVLVVAKVEKGKSKKLKS
VKELLGITIMERSSFEKNPIDFLEAKGYKEVKKDLIIKLPKYSLFELENGRKRMLASAGELQKGNELALP
SKYVNFLYLASHYEKLKGSPEDNEQKQLFVEQHKHYLDEIIEQISEFSKRVILADANLDKVLSAYNKHRD
KPIREQAENIIHLFTLTNLGAPAAFKYFDTTIDRKRYTSTKEVLDATLIHQSITGLYETRIDLSQLGGDG
SHTPGNSNKWKHLQENKKGRPRRGGSHTPGNSNKWKHLQENKKGRPRRGGSHTPGNSNKWKHLQENKKGR
PRRSLEVLFQ↓GPGSHHHHHH

Expression construct of 2x-Cas9(dS)-A22p3*:

MKSSHHHHHHGSSKSNEPGKATGEGKPVNNKWLNNAGKDLGSPVPDRIANKLRDKEFESFDDFRETFWEE
VSKDPELSKQFSRNNNDRMKVGKAPKTRTQDVSGKRTSFELNHQKPIEQNGGVYDMDNISVVTPKRNIDI

EGGGGGSLEVLFQ↓GPNATPKKRKVGGSPKKRKVGIHGVPAATMDKKYSIGLDIGTNSVGWAVITDEYKVP
SKKFKVLGNTDRHSIKKNLIGALLFDSGETAEATRLKRTARRRYTRRKNRI**S**YLQEIFSNEMAKVDDSFF
HRLEESFLVEEDKKHERHPIFGNIVDEVAYHEKYPTIYHLRKKLVDSTDKADLRLIYLALAHMIKFRGHF
LIEGDLNPDNSDVDKLFIQLVQTYNQLFEENPINASGVDAKAILSARLSKSRRLENLIAQLPGEKKNGLF
GNLIALSLGLTPNFKSNFDLAEDAKLQLSKDTYDDDLDNLLAQIGDQYADLFLAAKNLSDAILLSDILRV
NTEITKAPLSASMIKRYDEHHQDLTLLKALVRQQLPEKYKEIFFDQSKNGYAGYIDGGASQEEFYKFIKP
ILEKMDGTEELLVKLNREDLLRKQRTFDNGSIPHQIHLGELHAILRRQEDFYPFLKDNREKIEKILTFRI
PYYVGPLARGNSRFAWMTRKSEETITPWNFEEVVDKGASAQSFIERMTNFDKNLPNEKVLPKHSLLYEYF
TVYNELTKVKYVTEGMRKPAFLSGEQKKAIVDLLFKTNRKVTVKQLKEDYFKKIE**S**FDSVEISGVEDRFN
ASLGTYHDLLKIIKDKDFLDNEENEDILEDIVLTLTLFEDREMIEERLKTYAHLFDDKVMKQLKRRRYTG
WGRLSRKLINGIRDKQSGKTILDFLKSDGFANRNFMQLIHDDSLTFKEDIQKAQVSGQGDSLHEHIANLA
GSPAIKKGILQTVKVVDELVKVMGRHKPENIVIEMARENQTTQKGQKNSRERMKRIEEGIKELGSQILKE
HPVENTQLQNEKLYLYYLQNGRDMYVDQELDINRLSDYDVDHIVPQSFLKDDSIDNKVLTRSDKNRGKSD
NVPSEEVVKKMKNYWRQLLNAKLITQRKFDNLTKAERGGLSELDKAGFIKRQLVETRQITKHVAQILDSR
MNTKYDENDKLIREVKVITLKSKLVSDFRKDFQFYKVREINNYHHAHDAYLNAVVGTALIKKYPKLESEF
VYGDYKVYDVRKMIAKSEQEIGKATAKYFFYSNIMNFFKTEITLANGEIRKRPLIETNGETGEIVWDKGR
DFATVRKVLSMPQVNIVKKTEVQTGGFSKESILPKRNSDKLIARKKDWDPKKYGGFDSPTVAYSVLVVAK
VEKGKSKKLKSVKELLGITIMERSSFEKNPIDFLEAKGYKEVKKDLIIKLPKYSLFELENGRKRMLASAG
ELQKGNELALPSKYVNFLYLASHYEKLKGSPEDNEQKQLFVEQHKHYLDEIIEQISEFSKRVILADANLD
KVLSAYNKHRDKPIREQAENIIHLFTLTNLGAPAAFKYFDTTIDRKRYTSTKEVLDATLIHQSITGLYET
RIDLSQLGGDGSHTPGNSNKWKHLQENKKGRPRRGGSHTPGNSNKWKHLQENKKGRPRRGGSHTPGNSNK
WKHLQENKKGRPRR

sgRNA (for Cas9) or crRNA (for Cas12a) sequences:
spacer region, scaffold
Chemical modifications (AltR1/, /AltR2 modifications by IDT):
m – 2’-O methylation, * – phosphorothioate linkage

sgRNA targeting SV40 poly(A) transcription terminator in tdTomato assay (sgTom):
mA*mA*mG*UAAAACCUCUACAAAUGGUUUUAGAGCUAGAAAUAGCAAGUUAAAAUAAGGCUAGUCCGUU
AUCAACUUGAAAAAGUGGCACCGAGUCGG*mU*mG*mC

sgRNA targeting mGluR5:
mG*mG*mA*CUGACAGAAUCAACAGAGUUUUAGAGCUAGAAAUAGCAAGUUAAAAUAAGGCUAGUCCGUU
AUCAACUUGAAAAAGUGGCACCGAGUCGG*mU*mG*mC

sgRNA targeting TH:
mC*mC*mC*CAAGGUUCAUUGGACGGGUUUUAGAGCUAGAAAUAGCAAGUUAAAAUAAGGCUAGUCCGUU
AUCAACUUGAAAAAGUGGCACCGAGUCGG*mU*mG*mC

crRNA targeting SV40 poly(A) transcription terminator in tdTomato assay:
mU*mA*mA*UUUCUACUAAGUGUAGAUUUGCAGCUUAUAAUGGUUACA*mA*mA*mU
