# Supplementary material for: Engineering self-deliverable ribonucleoproteins for genome editing in the brain
Source: Nat Commun. 2024 Feb 26;15:1727. doi: 10.1038/s41467-024-45998-2 (PMC10897210; doi:10.1038/s41467-024-45998-2)
Supplement: Supplementary file 3 — Reporting Summary [file 41467_2024_45998_MOESM3_ESM.pdf]

nature portfolio

Corresponding author(s): Jennifer A. Doudna

Last updated by author(s): Jan 19, 2024

Reporting Summary

Nature Portfolio wishes to improve the reproducibility of the work that we publish. This form provides structure for consistency and transparency in reporting. For further information on Nature Portfolio policies, see our Editorial Policies and the Editorial Policy Checklist.

Statistics

For all statistical analyses, confirm that the following items are present in the figure legend, table legend, main text, or Methods section.

n/a	Confirmed	
☐	☒	The exact sample size (*n*) for each experimental group/condition, given as a discrete number and unit of measurement
☒	☐	A statement on whether measurements were taken from distinct samples or whether the same sample was measured repeatedly
☐	☒	The statistical test(s) used AND whether they are one- or two-sided<br>*Only common tests should be described solely by name; describe more complex techniques in the Methods section.*
☒	☐	A description of all covariates tested
☒	☐	A description of any assumptions or corrections, such as tests of normality and adjustment for multiple comparisons
☐	☒	A full description of the statistical parameters including central tendency (e.g. means) or other basic estimates (e.g. regression coefficient) AND variation (e.g. standard deviation) or associated estimates of uncertainty (e.g. confidence intervals)
☐	☒	For null hypothesis testing, the test statistic (e.g. *F*, *t*, *r*) with confidence intervals, effect sizes, degrees of freedom and *P* value noted<br>*Give P values as exact values whenever suitable.*
☒	☐	For Bayesian analysis, information on the choice of priors and Markov chain Monte Carlo settings
☒	☐	For hierarchical and complex designs, identification of the appropriate level for tests and full reporting of outcomes
☒	☐	Estimates of effect sizes (e.g. Cohen's *d*, Pearson's *r*), indicating how they were calculated

Our web collection on statistics for biologists contains articles on many of the points above.

Software and code

Policy information about availability of computer code

Data collection	Attune Cytometric Software (v5.1.1), Sony Cell Sorter Software (v2.1.5), Zetasizer (v7.13), Echo Pro (v6.4.2), CFX96 Touch Real-Time PCR System (BioRad), Thermo Scientific Invitrogen Countess 3 Automated Cell Counter.
Data analysis	NGS data were analyzed using CRISPResso2 (http://crispresso.pinellolab.org/submission). Attune Cytometric Software (v5.1.1), FlowJo (v10.7.1), Spangene (v4.3.6), Excel (Microsoft 365), QuPath software (v.0.3.2), Prism 9 (GraphPad Software v.9.4.1) ZEN3.8 (Carl Zeiss Microscopy GmbH), and Snapgene (v4.3.6), were used for the analysis of other data.

For manuscripts utilizing custom algorithms or software that are central to the research but not yet described in published literature, software must be made available to editors and reviewers. We strongly encourage code deposition in a community repository (e.g. GitHub). See the Nature Portfolio guidelines for submitting code & software for further information.

Data

Policy information about availability of data

All manuscripts must include a data availability statement. This statement should provide the following information, where applicable:

- Accession codes, unique identifiers, or web links for publicly available datasets
- A description of any restrictions on data availability
- For clinical datasets or third party data, please ensure that the statement adheres to our policy

Protein, peptide, and RNA sequences in this study are available in the supplementary materials. Plasmid sequences, sequencing data, and raw images are available

through Dryad (DOI: 10.5061/dryad.mkkwh716m). Next-generation sequencing data are available on NCBI (PRJNA1073688). Source data are provided with this paper. Relevant materials (e.g., plasmids, proteins) are available from the corresponding author upon reasonable request or from Addgene.

Research involving human participants, their data, or biological material

Policy information about studies with human participants or human data. See also policy information about sex, gender (identity/presentation), and sexual orientation and race, ethnicity and racism.

Reporting on sex and gender	N/A.
Reporting on race, ethnicity, or other socially relevant groupings	N/A.
Population characteristics	N/A.
Recruitment	N/A.
Ethics oversight	N/A.

Note that full information on the approval of the study protocol must also be provided in the manuscript.

Field-specific reporting

Please select the one below that is the best fit for your research. If you are not sure, read the appropriate sections before making your selection.

☒ Life sciences ☐ Behavioural & social sciences ☐ Ecological, evolutionary & environmental sciences

For a reference copy of the document with all sections, see nature.com/documents/nr-reporting-summary-flat.pdf

Life sciences study design

All studies must disclose on these points even when the disclosure is negative.

Sample size	No sample size calculation was performed for our study. All experiments were performed with 4-6 biological replicates. 100 cells or more were counted for each microscopy experiments. Sample sizes were determined based on preliminary experimental results and data variation and the standard design used in the related studies of the field or from the lab (e.g., doi: 10.1016/j.ymthe.2023.06.019).
Data exclusions	No data were excluded. Experiments that failed to generate reliable data due to technical issues (e.g., unsuccessful brain injections) were excluded from the final data analysis.
Replication	Cell experiments were performed with four biological replicates; animal experiments were performed with six biological replicates. Data were collected and analyzed from all these experimental replicates.
Randomization	For animal experiments, mice were randomized by cage and littermates. No further randomization was applied to the experiments.
Blinding	Blinding is irrelevant to the experiments due to the experimental settings described in Methods and figure legends.

Reporting for specific materials, systems and methods

We require information from authors about some types of materials, experimental systems and methods used in many studies. Here, indicate whether each material, system or method listed is relevant to your study. If you are not sure if a list item applies to your research, read the appropriate section before selecting a response.

Materials & experimental systems

n/a	Involved in the study	
☐	☒	Antibodies
☐	☒	Eukaryotic cell lines
☒	☐	Palaeontology and archaeology
☐	☒	Animals and other organisms
☒	☐	Clinical data
☒	☐	Dual use research of concern
☒	☐	Plants

Methods

n/a	Involved in the study	
☒	☐	ChIP-seq
☐	☒	Flow cytometry
☒	☐	MRI-based neuroimaging

Antibodies

Antibodies used	Anti-NeuN (Millipore Sigma, CAT# ABN78)
Validation	The primary antibody used in the manuscript was validated based on the supplier statement (https://www.emdmillipore.com/US/en/product/Anti-NeuN-Antibody-rabbit,MM_NF-ABN78?ReferrerURL=https%3A%2F%2Fwww.google.com%2F#anchor_Product%20Information).

Eukaryotic cell lines

Policy information about cell lines and Sex and Gender in Research

Cell line source(s)	Mouse neural progenitor cells (NPCs) isolated from cortices from Embryonic Day 13.5 Ai9-tdTomato homozygous mouse embryos (male or female). HEK293T-GFP obtained from UC Berkeley Cell Culture Facility.
Authentication	Cells were authenticated by the suppliers or the Doudna lab, and the key transgenes (stop cassette-tdTomato and EGFP trangenes) targeted for genome editing purposes were PCR- and sequencing-confirmed with corresponding primers.
Mycoplasma contamination	Cell lines were confirmed to be mycoplasma-free.
Commonly misidentified lines (See ICLAC register)	No commonly misidentified cell lines were used.

Animals and other research organisms

Policy information about studies involving animals; ARRIVE guidelines recommended for reporting animal research, and Sex and Gender in Research

Laboratory animals	Ai9 and wild-type mice (Jackson Laboratory, Bar Harbor, ME) were group-housed at the University of California, Berkeley, with a 12-h light-dark cycle and allowed to feed and drink ad libitum, at 20-22 °C with 40-60% humidity, according to the supplier's instruction (www.jax.org/jax-mice-and-services/customer-support/technical-support/breeding-and-husbandry-support/mouse-room-conditions). Ai9 and wild-type mice aged 8-12 weeks were used for the study.
Wild animals	No wild animals were used in the study.
Reporting on sex	Ai9 and wild-type mice (male or female, 8-12 weeks) were used. The findings in the study are not sex-specific.
Field-collected samples	No field collected samples were used in the study.
Ethics oversight	Housing, maintenance, and experimentation of the mice were carried out with strict adherence to ethical regulations set forth by the Animal Care and Use Committee (ACUC) at the University of California, Berkeley.

Note that full information on the approval of the study protocol must also be provided in the manuscript.

Plants

Seed stocks	N/A.
Novel plant genotypes	N/A.
Authentication	N/A.

Flow Cytometry

Plots

Confirm that:

☒ The axis labels state the marker and fluorochrome used (e.g. CD4-FITC).

☒ The axis scales are clearly visible. Include numbers along axes only for bottom left plot of group (a 'group' is an analysis of identical markers).

☒ All plots are contour plots with outliers or pseudocolor plots.

☒ A numerical value for number of cells or percentage (with statistics) is provided.

Methodology

Sample preparation	NPCs or HEK293T cells were trypsinized, quenched in medium, pelleted, resuspended in PBS, and filtered through a 40-micron cell strainer prior to flow cytometry or FACS.
Instrument	Attune NxT acoustic focusing cytometer (Thermo Fisher Scientific); Sony Cell Sorter SH800Z
Software	Attune Cytometric Software v5.1.1; Sony Cell Sorter Software v2.1.5.
Cell population abundance	tdTomato-positive or GFP-positive cells were sorted with an efficiency of >95% prior to analyses.
Gating strategy	1) FSC-A/SSC-A used to remove debris; 2) FSC-A/FSC-H used to define cell singlets; 3) FSC-A/GFP-A, RFP-A used to determine fluorescence. Gating for RFP-positive cells (upon editing) was based on RFP-negative cells (unedited or edited using non-targeting sgRNA). GFP knockdown was based on GFP-positive cells (unedited or edited using non-targeting sgRNA).

☒ Tick this box to confirm that a figure exemplifying the gating strategy is provided in the Supplementary Information.
